# Comparative transcriptome profiling of selected osmotic regulatory proteins in the gill during seawater acclimation of chum salmon (*Oncorhynchus keta*) fry

**DOI:** 10.1038/s41598-020-58915-6

**Published:** 2020-02-06

**Authors:** Sang Yoon Lee, Hwa Jin Lee, Yi Kyung Kim

**Affiliations:** 1 The East Coast Research Institute of Life Science, Gangneung-Wonju National University, Gangneung, 25457 South Korea; 2 Department of Marine Biotechnology, Gangneung-Wonju National University, Gangneung, 25457 South Korea

**Keywords:** Transport carrier, Transport carrier, Transcriptomics, Transcriptomics, Transcriptomics

## Abstract

Salmonid fishes, chum salmon (*Oncorhynchus keta*) have the developed adaptive strategy to withstand wide salinity changes from the early life stage. This study investigated gene expression patterns of cell membrane proteins in the gill of chum salmon fry on the transcriptome level by tracking the salinity acclimation of the fish in changing environments ranging from freshwater (0 ppt) to brackish water (17.5 ppt) to seawater (35 ppt). Using GO analysis of DEGs, the known osmoregulatory genes and their functional groups such as ion transport, transmembrane transporter activity and metal ion binding were identified. The expression patterns of membrane protein genes, including pump-mediated protein (NKA, CFTR), carrier-mediated protein (NKCC, NHE3) and channel-mediated protein (AQP) were similar to those of other salmonid fishes in the smolt or adult stages. Based on the protein-protein interaction analysis between transmembrane proteins and other related genes, we identified osmotic-related genes expressed with salinity changes and analyzed their expression patterns. The findings of this study may facilitate the disentangling of the genetic basis of chum salmon and better able an understanding of the osmophysiology of the species.

## Introduction

Salinity is one of the critical factors limiting the distribution patterns of all aquatic organisms^1–4^. Salmonid fishes display diverse life-history traits; anadromous individuals that mature in the river from hatching through to juveniles acquire the capacity to tolerate salinity associated with parr–smolt transformation and undergo ocean migrations before returning to rivers for spawning, whereas landlocked types spend their entire life within freshwater^5,6^. Although migration between habitats is common among salmonid fishes, the seawater acclimation period varies even within anadromous species. Therefore, the timing of river to ocean migration varies from species to species^5,7,8^.

Chum salmon (*Oncorhynchus keta*) possess an excellent osmotic plasticity in coping with hyperosmotic or hypoosmotic environments^9–11^. During the late embryonic stage, chum salmon have already acquired the hypo-osmoregulatory mechanism by the mitochondria-rich cells (MRCs) in the yolk-sac membrane^12^. In addition, chum salmon fry whose habitat is freshwater begin to show remarkable seawater adaptability prior to seawater entry, which is not observed in the fry of other salmonids^13^. Chum salmon begin to activate MRCs in the gill at an earlier stage (alevin-fry) and show higher salinity resistance at the fry stage than at the late alevin stages^11,14,15^.

Most of the salmonid fishes currently in the market are dominated by cultured Atlantic salmon (*Salmo salar*), whereas the production of chum salmon mostly depends on fishing (FAO, 2019). It is also noteworthy that chum salmon have been studied less when compared to other salmonid fishes. However, chum salmon are a major species of salmonid fishes that return to Korea, and if the feed and the aquaculture system are improved with the help of research on seawater adaptability and growth, they can be developed as a promising aquaculture species in the future.

The gill is a primary organ to detect changes in external osmotic pressure and promotes the compensatory active absorption or the excretion of monovalent ions (sodium, potassium, chloride) to maintain the osmolality of body fluids at levels equivalent to approximately one-third of the seawater osmolality^16–18^. In seawater, the fish must drink copious amount of seawater and absorb water in the intestines to compensate for the loss of water, while the excess ions are actively excreted from the gills and the kidney^19^. In contrast, freshwater teleost cope with the osmotic water load and the ion loss and therefore excrete a large amount of water by producing diluted urine by the kidney and uptake ions through the gills^19^. In addition, the gill tissues play crucial roles in physical processes such as gas exchange, nitrogenous waste excretion, and acid-base balance^20^.

To accomplish these functions implicated in the maintenance of domestic homeostasis, various membrane proteins of ionocytes regulate intracellular ionic concentrations^21^. Ionocytes also regulate acid (H^+^) or base (HCO_3_^−^) release to maintain pH homeostasis in the blood^21,22^. In the euryhaline species, the plasma osmolality was reduced when the seawater adapted fishes (*Acanthopagrus schlegeli* and *Paralichthys orbignyan*) were transferred to freshwater^23,24^. In contrast, it has been reported that the plasma osmolality increased when freshwater-adapted fishes (*Takifugu obscurus, Synechogobius ommaturus* (R.), and *Chanos chanos*) were moved to brackish-water or seawater^25–27^. In the diadromous species (*Monopterus albus, S. salar*), the plasma osmolality increased when freshwater-adapted individuals were transported to seawater and the plasma osmolality was gradually decreased during long-term exposure and then was maintained at a certain level^28,29^. Osmotic control of gill ionocytes is known to involve various membrane proteins such as sodium-potassium ATPase (NKA), sodium-potassium-chloride cotransporter (NKCC), cystic fibrosis transmembrane conductance regulator (CFTR) and aquaporin (AQP)^30–32^. In addition, for research on osmoregulation capabilities of euryhaline fish, it is important to investigate not only the gene expression patterns of transmembrane proteins but also those of interrelated proteins under the influence of salinity stress.

Studies on various aspects of seawater adaptation and osmotic control capacity of salmonid fishes have long been reported. However, a molecular genetic approach to addressing osmotic-related genes involved the osmoregulation and the maintenance of the body homeostasis under salinity changes has rarely been implemented. In addition, studies of the transcriptome level of osmolality-regulating proteins have been rarely reported. Therefore, a deeper understanding of the mechanism underlying the adaptation to salinity stress of the chum salmon may contribute to developing strategies for efficient farming practices for this candidate species. In that regard, this study tried to analyze gene expression patterns of membrane proteins present in ionocytes and a gill tissue with whole transcriptome NGS and qRT-PCR. In addition to that, the gene expression of related proteins was studied as well.

## Results

### Genome mapping and *de-novo* assembly of unmapped reads

The reads obtained from sequencing of each group were trimmed and deposited at Genbank under the Sequence Read Archive (SRA) accessions SRX3932910-SRX3932912. Since chinook salmon are taxonomically close to chum salmon, each group showed a high read-mapping rate of 82 to 83% in Table S1^6,33^. An additional *de-novo* assembly was conducted on the unmapped reads from the chinook salmon genome assembly, thereby constructing 119,439 counts of N50, 403 bp. Subsequently, a new reference of 197,684 counts (N50 length: 3154 bp, Avr length: 1,428 bp, GC contents: 48%) were constructed by pooling the reads from both the chinook salmon genome assembly and the reads from the *de-novo* assembly. Finally, the reads of each group were mapped to the newly constructed reference (Table 1). The mapping rate of the new reference was 94–95% and the average length was 1428.82 bp.

**Table 1:** Mapping statistics of transcriptome reads to the reconstructed transcriptome assembly.

	Freshwater (0%)		Brackish water (50%)		Seawater (100%)	
	No. reads	Rate (%)	No. reads	Rate (%)	No. reads	Rate (%)
Mapped reads	91,480,087	94.14	92,338,752	94.45	105,031,902	94.37
Not mapped reads	5,697,147	5.86	5,424,666	5.55	6,268,988	5.63
Reads in pairs	73,700,972	75.84	74,482,876	76.19	84,457,112	75.88
Broken paired reads	17,779,115	18.30	17,855,876	18.26	20,574,790	18.49
Total reads	97,177,234	100.00	97,763,418	100.00	111,300,890	100.00

### Functional annotation of reconstructed reference reads

Annotation was conducted on the reads mapped to the newly constructed reference based on a variety of databases. The BLAST annotation based on the NR database identified that a total of 93,542 counts (47.31%) were matched with certain genes. Most of the matched genes originated from chinook salmon (*O. tshawytscha*), which were used as a reference, and the rest of the annotated genes were mostly related to the salmon origin group: rainbow trout (*O. mykiss*, 14.9%), coho salmon (*O. kisutch*, 8.3%), Atlantic salmon (*S. salar*, 6.1%) and Arctic char (*Salvelinus alpinus*, 3.2%) (Fig. S1). GO annotation was carried out on three categories (biological process; BP, molecular function; MF, cellular component; CC) under the condition of level 7, and the results were merged with those of the annotation analyses of Interpro, GO-Slim and EggNog. In the category of BP, the most frequently annotated GO classes were nucleic acid-templated transcription (GO: 0097659) and regulation of nucleic acid-templated transcription (GO: 1903506). Among those, 6,806 reads and 6,469 reads were mapped, respectively (Fig. S2). In the case of the MF category, the genes related to nucleoside-triphosphatase activity (GO: 0017111) and zinc ion binding (GO: 0008270) were annotated frequently, more than any other gene with 2,928 reads and 2,613 reads mapped. As for the CC category, the genes responsible for clathrin-coated vesicle (GO: 0030136) were annotated the most with 342 reads mapped.

### Clustering analysis of differential gene expression pattern

**Figure 1:**
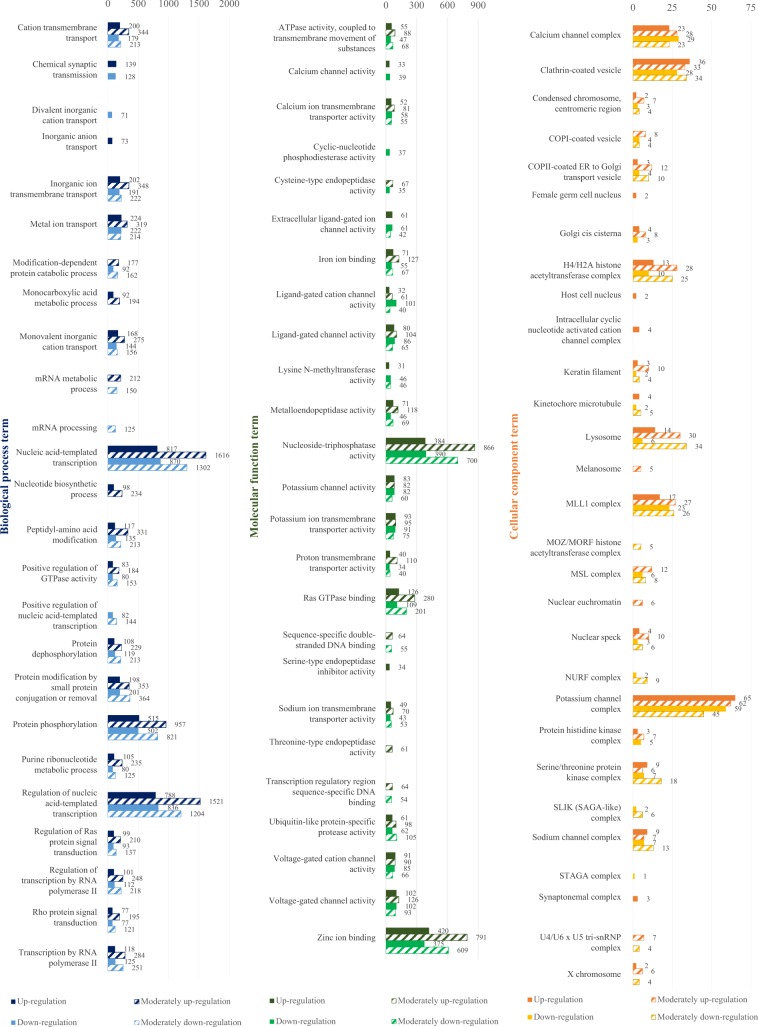
Gene ontology annotation (by level 7) for functional analysis of differentially expressed genes of *O. keta* fry after transfer from freshwater to brackish water.

**Figure 2:**
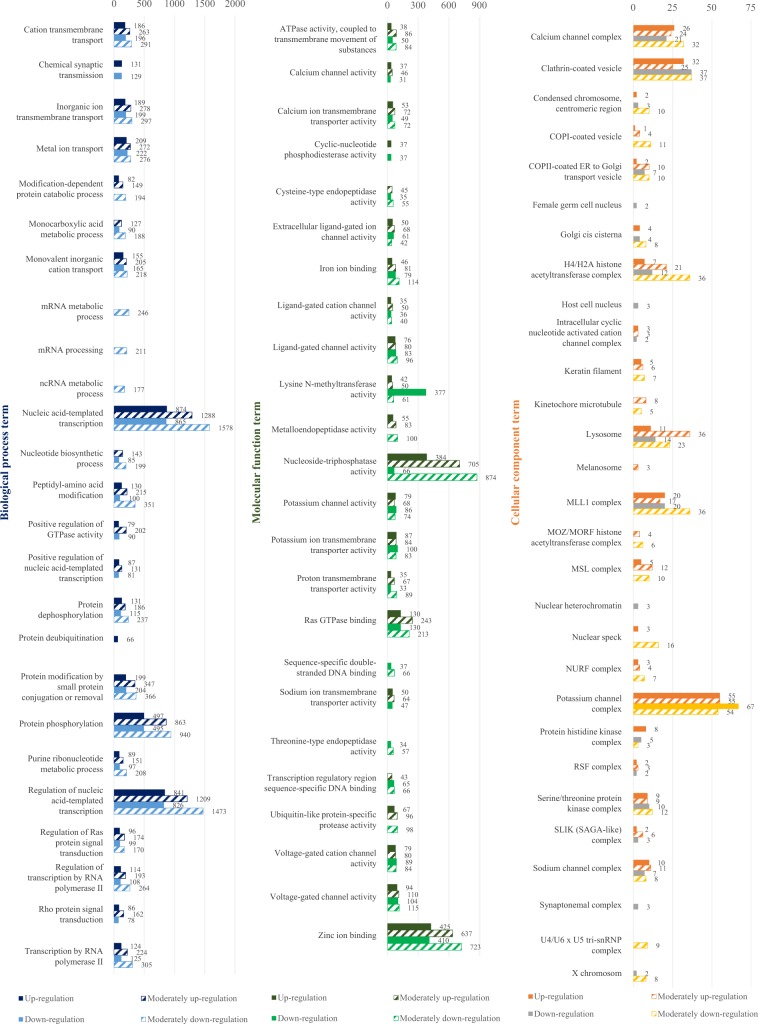
Gene ontology annotation (by level 7) for functional analysis of differentially expressed genes of *O. keta* fry after transfer from brackish water to seawater.

DEGs were investigated using transcriptome profiles of gills of chum salmon fry in different salinity environments. Among the pump-mediated transmembrane proteins which are directly involved in osmoregulation, NKA (ATP1a, ATP1b) is known to be associated with metal ion transport (GO: 0030001) and inorganic ion transmembrane transport (GO: 0098660) in BP term, and potassium ion transmembrane transporter activity (GO: 0071805) in MF term (Figs. 1 and 2). Along with ATP1a and ATP1b, the genes involved in metal ion transport, inorganic ion transmembrane transport and potassium ion transmembrane transporter activity showed the pattern of gene expression with chum salmon fry during the environmental alteration: when the chum salmon were transferred from freshwater to brackish water, 1.14 times more genes were expressed than when the fish were transferred from brackish water to seawater. Conversely, when the fish were transferred from brackish water to seawater, there was a 1.16-fold increase in the number of the genes that decreased their expression compared to when the fish were transferred from freshwater to brackish water. NKCC (SLC12a, SLC9a), the carrier-mediated transmembrane protein, is associated with cation transmembrane transport (GO: 0098655), metal ion transport (GO: 0030001), metal ion transport (GO: 0030001), monovalent inorganic cation (GO: 0015672) and inorganic ion transmembrane transport (GO: 0098660) in BP term, and in MF term it is involved in sodium ion transmembrane transporter activity (GO: 0015081) and proton transmembrane transporter (GO: 0015078). The genes involved in cation transmembrane transport, monovalent inorganic cation and proton transmembrane transporter also showed the gene expression pattern: when transferred from freshwater to brackish water, chum salmon fry tended to express more genes than when transferred from brackish water to seawater. The genes were expressed less when the fish were transferred from brackish water to seawater than when transferred from freshwater to brackish water. In addition, there was a tendency of the expression of the genes involved in iron ion binding (GO: 0005506), potassium channel activity (GO: 0008282), and potassium channel complex (GO: 0034705) to increase more remarkably when the chum salmon fry were transferred from freshwater to brackish water than when transferred from brackish water to seawater. In particular, 1.56 times more genes were expressed which were involved in iron ion binding (GO: 0005506) when the salinity was increased from freshwater to brackish water than from brackish water to seawater. The number of genes involved in the potassium channel activity (GO: 0008282), and potassium channel complex (GO: 0034705) was about 1.12 times and 1.15 times, respectively.

**Figure 3:**
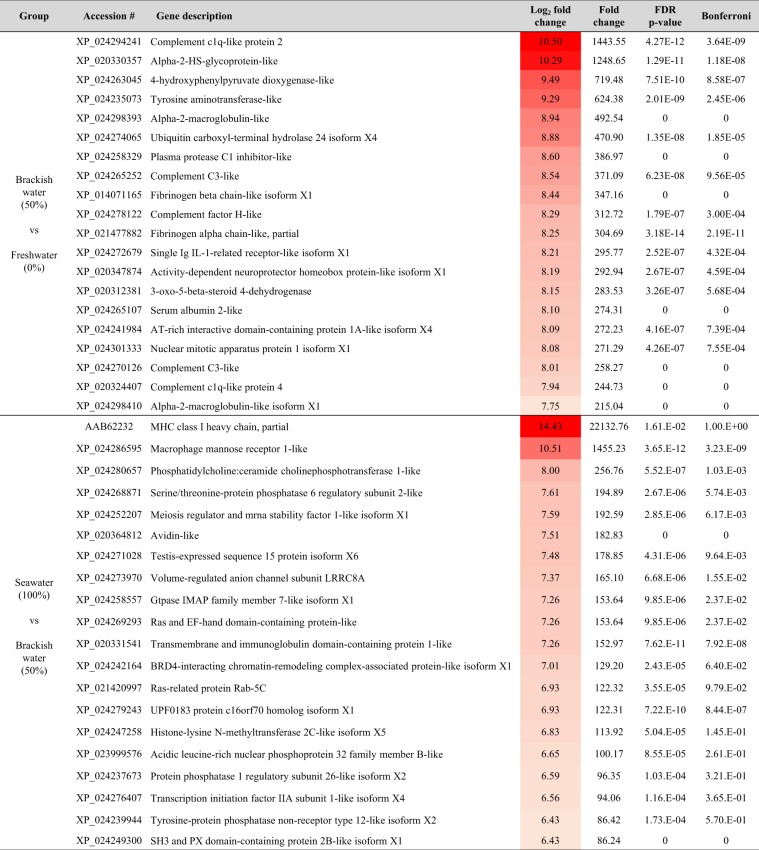
List of the first 20 genes showing the highest differential expression in salinity changes for the gill of *O. keta* fry.

The genes showing an increase of expression in both groups, from freshwater to brackish water group and from brackish water to seawater group, were listed in order of fold change. As a result, the types of genes differed between the two groups but showed similar trends in function (Fig. 3). In the salinity change from freshwater to brackish water, an increase in the expression of genes involved in innate immune response and blood coagulation was noticeable. Changes in salinity from brackish water to seawater increased the expression of genes involved in adaptive immunity. In terms of cellular components, the genes involved in binding to the integral component of membrane or cell surface and metal ion binding were expressed in both groups.

The enumeration of the genes in fold change order which were highly subject to salinity changes indicated that the types of genes were different between the freshwater to brackish water transfer group and the brackish water to seawater transfer group. However, the functions and the locations of the genes of the two groups showed many similarities (Fig. 3). The following proteins located either in the integral component of the membrane or in the cell surface showed high differential gene expression: single Ig IL-1-related receptor-like isoform X1, 3-oxo-5-beta-steroid 4-dehydrogenase, MHC class I heavy chain, macrophage mannose receptor 1-like and phosphatidylcholine: ceramide cholinephosphotransferase 1-like, serine/threonine-protein phosphatase 6 regulatory subunit 2-like, transmembrane and immunoglobulin domain-containing protein 1-like. Also, the genes involved in metal ion binding, including 4-hydroxyphenylpyruvate dioxygenase-like, activity-dependent neuroprotector homeobox protein-like isoform X1 and histone-lysine N-methyltransferase 2C-like isoform X5 showed high differential gene expression as the salinity increased. However, plasma protease C1 inhibitor-like, fibrinogen beta chain-like isoform X1 and fibrinogen alpha chain-like which were associated with blood coagulation showed unusually high differential gene expression when the fish were transferred from freshwater to brackish water.

### PPI networks of osmoregulation related genes

**Figure 4:**
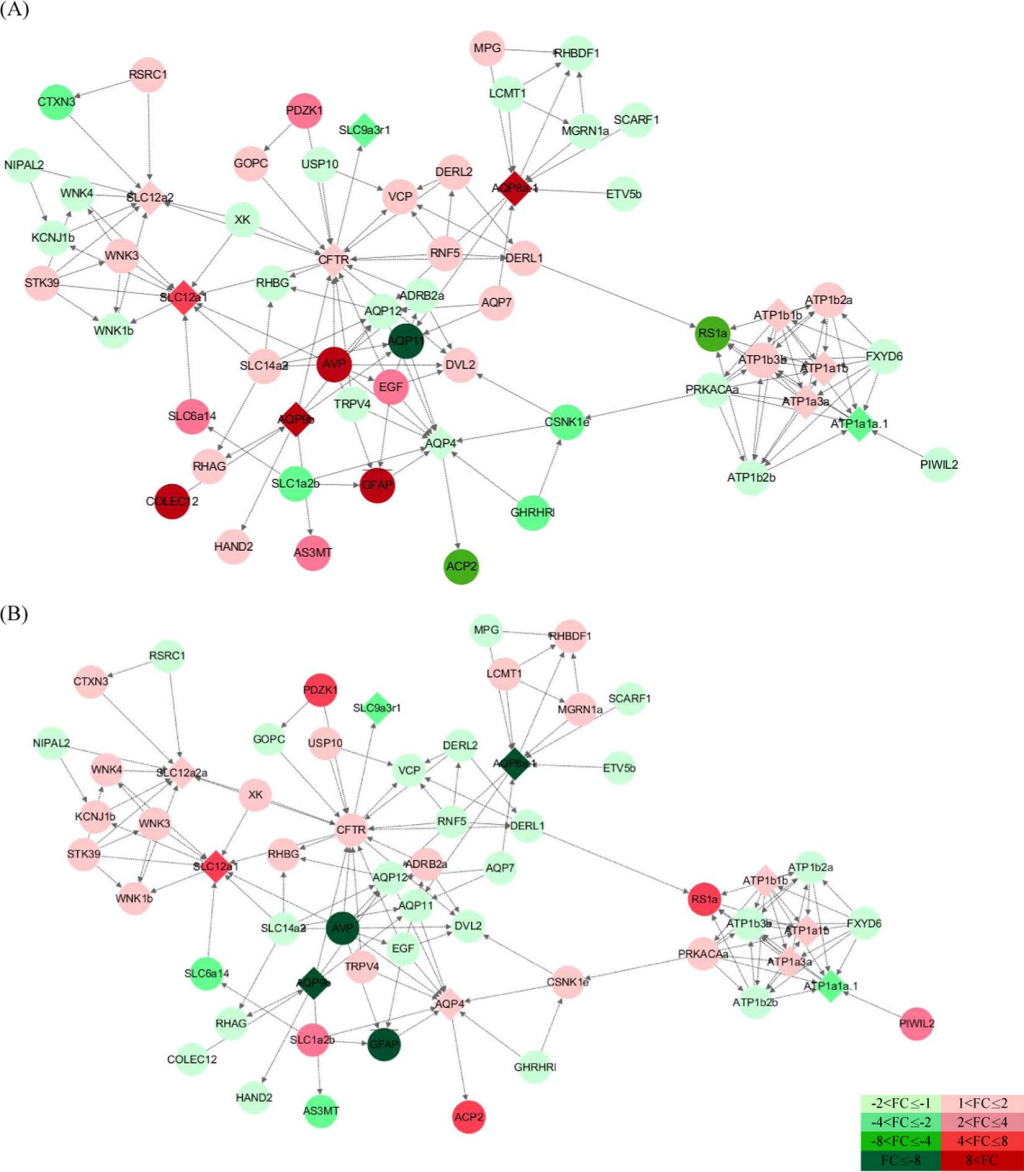
PPI network map of osmoregulation-related proteins using STRING. The red-colored figure (light to dark) represents the up-regulated protein and the green-colored figure (light to dark) represents the down-regulated protein. The saturation is displayed differently according to fold change (FC). The diamond shape is the main transmembrane protein in this study and the circle shape is a protein interacting with a transmembrane protein. (**A**) illustrates the difference in expression between the brackish water vs freshwater group, and (**B**) illustrates the difference in expression between the seawater vs brackish water group.

The interaction network of osmoregulatory proteins provides important information about homeostasis responses of fish to salinity changes. PPI network analyses on a total of 59 nodes showed that the genes tended to be grouped according to the functions of the membrane protein genes and each protein was interrelated with each other (Fig. 4). The PPI map consisted of a total of 138 edges and the average local clustering coefficient was 0.638. The average node degree was 4.68 and the PPI enrichment p-value was below 1.0e-16. The functional enrichment analyses of PPI networks indicated that Reactome Pathways was involved in the pathways of transport of small molecules (DRE-382551), ion homeostasis (DRE-5578775), ion transport by P-type ATPases (DRE-936837), aquaporin-mediated transport (DRE-445717) and passive transport by aquaporins (DRE-432047).

The domain and keyword analyses using Uniprot, PFAM, INTERPRO and SMART confirmed the aforementioned finding. As the salinity increased, the membrane protein genes and the interaction proteins of chum salmon fry were variously expressed (Table 2). However, in the gill of chum salmon fry, the genes interacting with membrane proteins were commonly present in the pattern of alternating increases and decreases in expression, rather than a continuous increase or decrease in expression with increasing salinity.

**Table 2:** List of proteins interacting with transmembrane proteins present in the STRING database and differences in expression between each group.

Gene (*Danio rerio*)	Symbol	Accession # (*O. tshawytscha*)	Fold change	
			50% vs 0%	100% vs 50%
Acid phosphatase 2, lysosomal	ACP2	XM_024439857.1	−4.64	5.84
Adrenoceptor beta 2, surface a	ADRB2a	XM_024393505.1	−1.50	1.21
Aquaporin 4	AQP4	XM_024375186.1	−1.63	1.73
Aquaporin 7	AQP7	XM_024401632.1	1.85	−1.67
Aquaporin 8a.1	AQP8a.1	XM_024433576.1	743.33	−13.62
Aquaporin 9b	AQP9b	XM_024440258.1	100.44	−21.22
Aquaporin 11	AQP11	XM_024445684.1	−8.88	−1.01
Aquaporin 12	AQP12	XM_024421535.1	−1.17	−1.01
Arginine vasopressin	AVP	XM_024397535.1	27.90	−26.48
Arginine/serine-rich coiled-coil 1	RSRC1	XR_002955720.1	1.14	−1.23
Arsenite methyltransferase	AS3MT	XM_024403019.1	3.13	−3.85
ATPase Na+/K+ transporting subunit alpha 1a	ATP1a1a	XM_024412034.1	−2.47	−2.69
ATPase Na+/K+ transporting subunit alpha 1b	ATP1a1b	XM_024443742.1	1.08	1.34
ATPase Na+/K+ transporting subunit alpha 1c	ATP1a1c	XM_024443741.1	−1.00	1.11
ATPase Na+/K+ transporting subunit alpha 3	ATP1a3	XM_024441658.1	1.13	1.07
ATPase Na+/K+ transporting subunit beta 1	ATP1b1	XM_024408556.1	1.14	1.18
ATPase Na+/K+ transporting subunit beta 2a	ATP1b2a	XM_024394116.1	1.61	−1.04
ATPase Na+/K+ transporting subunit beta 2b	ATP1b2b	XM_024394118.1	−1.24	−1.27
ATPase Na+/K+ transporting subunit beta 3b	ATP1b3b	XM_024376682.1	1.11	−1.28
Camp-dependent protein kinase catalytic subunit alpha	PRKACAa	XM_024390584.1	−1.63	1.21
Casein kinase 1, epsilon	CSNK1e	XM_024379721.1	−2.00	1.83
Collectin sub-family member 12	COLEC12	XM_024393027.1	358.79	−1.56
Cortexin 3	CTXN3	XM_024381564.1	−3.49	1.63
Cystic fibrosis transmembrane conductance regulator	CFTR	XM_024424084.1	1.16	1.29
Derlin 1	DERL1	XM_024405756.1	1.04	−1.10
Derlin 2	DERL2	XM_024393885.1	1.11	−1.05
Dishevelled segment polarity protein 2	DVL2	XM_024376794.1	1.29	−1.90
Epidermal growth factor	EGF	XM_024390462.1	2.55	−1.70
Ets variant 5b	ETV5b	XM_024389695.1	−1.34	−1.26
FXYD domain containing ion transport regulator 6	FXYD6	XM_024442135.1	−1.30	−1.35
Glial fibrillary acidic protein	GFAP	XM_024385320.1	18.95	−17.99
Golgi-associated PDZ and coiled-coil motif containing	GOPC	XM_024378000.1	1.52	−1.63
Growth hormone releasing hormone receptor, like	GHRHRl	XM_024422521.1	−2.19	−1.39
Heart and neural crest derivatives expressed 2	HAND2	XM_024436576.1	1.37	−1.36
Leucine carboxyl methyltransferase 1	LCMT1	XM_024387973.1	−1.23	1.31
Mahogunin, ring finger 1a	MGRN1a	XM_024433250.1	−1.04	1.44
NIPA like domain containing 2	NIPAL2	XR_002952192.1	−1.02	−1.02
N-methylpurine DNA glycosylase	MPG	XM_024433243.1	1.15	−1.47
PDZ domain containing 1	PDZK1	XM_024409391.1	3.73	5.43
Piwi-like RNA-mediated gene silencing 2	PIWIL2	XM_024417801.1	−1.26	2.46
Potassium inwardly-rectifying channel, subfamily J, member 1b	KCNJ1b	XM_024445818.1	−1.42	1.30
Retinoschisin 1a	RS1a	XM_024422637.1	−4.34	4.69
Rh associated glycoprotein	RHAG	XM_024377707.1	1.23	−1.15
Rh family, B glycoprotein (gene/pseudogene)	RHBG	XM_024437515.1	−1.22	1.35
Rhomboid 5 homolog 1a	RHBDF1	XM_024387965.1	−1.09	1.01
Ring finger protein 5	RNF5	XM_024396104.1	1.15	−1.06
Scavenger receptor class F, member 1	SCARF1	XM_024445009.1	−1.15	−1.14
Serine threonine kinase 39	STK39	XM_024388934.1	1.52	1.52
Solute carrier family 1 member 2b	SLC1a2b	XM_024413392.1	−2.06	2.03
Solute carrier family 6 member 14	SLC6a14	XM_024427745.1	2.53	−2.25
Solute carrier family 9 member 3	SLC9a3	XM_024396240.1	−2.00	−2.22
Solute carrier family 12 member 1	SLC12a1	XM_024440802.1	7.02	4.87
Solute carrier family 12 member 2a	SLC12a2a	XM_024381566.1	1.48	1.26
Solute carrier family 12 member 2b	SLC12a2b	XM_024418405.1	1.28	1.56
Solute carrier family 14 member 2	SLC14a2	XM_024417716.1	1.27	−1.34
Transient receptor potential cation channel, subfamily V, member 4	TRPV4	XM_024417109.1	−1.09	1.07
Ubiquitin specific peptidase 10	USP10	XM_024440227.1	−1.56	1.82
Valosin containing protein	VCP	XM_024381824.1	1.08	−1.25
WNK lysine deficient protein kinase 1b	WNK1b	XM_024425441.1	−1.09	1.10
WNK lysine deficient protein kinase 3	WNK3	XM_024384949.1	1.02	1.25
WNK lysine deficient protein kinase 4	WNK4	XM_024388112.1	−1.28	1.55
linked Kx blood group (mcleod syndrome)	XK	XM_024409767.1	−1.84	1.79

### qRT-PCR validation of transmembrane protein genes related to osmoregulation

**Figure 5:**
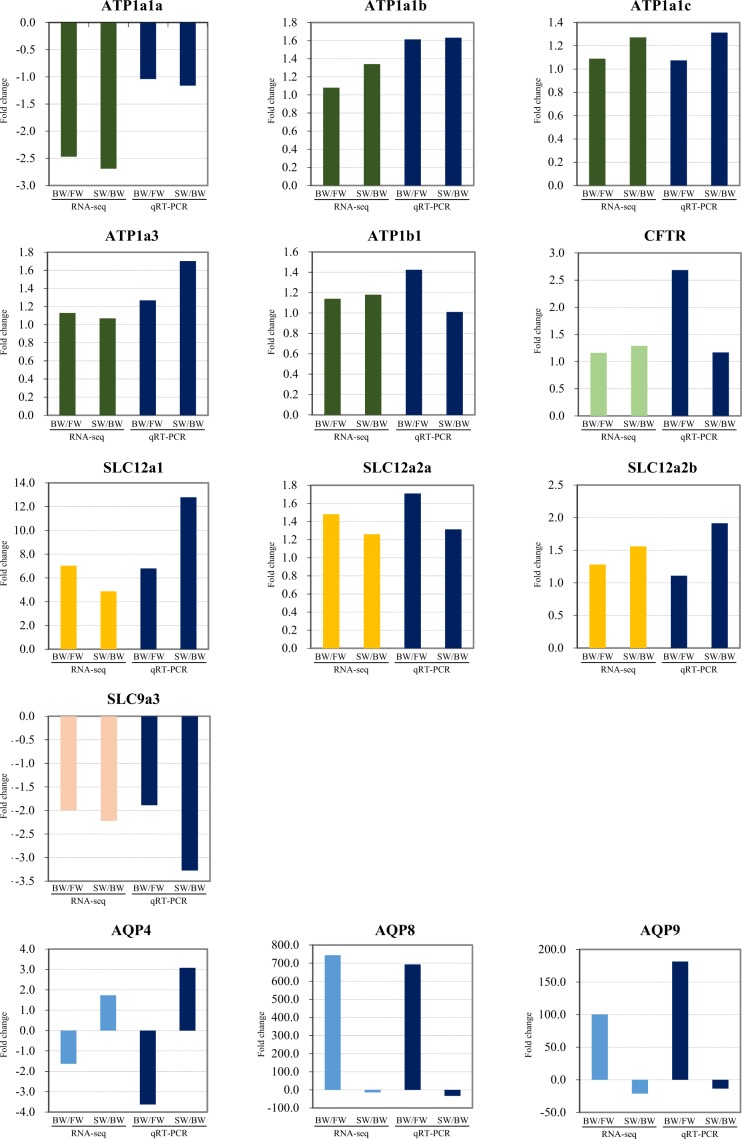
Validation of the RNA-seq data by qRT-PCR. Target genes were normalized to the reference gene, elongation factor 1 alpha. FW, freshwater; BW, brackish water; SW, seawater.

To confirm the expression patterns of DEGs, representative transmembrane protein genes were selected for qRT-PCR analysis. As shown in Fig. 5, most of the qRT-PCR results of the genes analyzed kept consistent with the high-throughput sequencing data, which confirmed the accuracy and reliability of the sequencing data. Based on qRT-PCR, mRNA expression levels of ATP1a1b, ATP1a1c, ATP1a3, ATP1b1 and CFTR related to pump mediated ion transport in the brackish water vs. freshwater group were almost 1.61-, 1.07-, 1.27-, 1.42 and 2.68 -fold of the control respectively, and those in the seawater vs brackish water group were almost 1.63-, 1.31-, 1.70-, 1.01 and 1.17-fold of the control respectively, the expression tended to increase with increasing salinity. On the other hand, in the case of ATP1a1a, as the salinity increased, gene expression tended to decrease by 1.04- and 1.16-fold. As for SLC12a2a and SLC12a2b, a carrier-mediated symporter, gene expression slightly increased in both the brackish water vs. freshwater and the seawater vs. brackish water group. However, SLC12a1 had the fold change value four to five times higher than that of SLC12a2a and SLC12a2b in the two comparative groups. The SLC9a3, a carrier-mediated antiporter, showed a similar pattern to ATP1a1a in which gene expression decreased with the increasing salinity. In the case of AQP, a channel-mediated protein, the fold change value as well as the expression patterns of the two groups differ from each other according to the isoform type. Based on the mRNA expression level results of qRT-PCR, the following was found: AQP4 showed decreased gene expression in the brackish water vs 0% group by 3.63-fold and increased gene expression in the seawater vs. brackish water group by 3.08-fold. AQP8 and AQP9 showed increased expression in the brackish water vs. freshwater group by 692.98- and 181.44-fold, respectively, and decreased expression in the seawater vs brackish water group by 32.75- and 13.42-fold, respectively.

## Discussion

Chum salmon, the excellent osmoregulator, migrate downstream to the sea in their early life stage, acquiring hypo-osmoregulatory capability during the alevin stages. To further examine the potential mechanisms and identify osmotic-regulated genes, we compared the transcriptome of chum salmon gill tissues of two groups: those transferred from freshwater to brackish water and from brackish water to sea water. Among the membrane proteins associated with osmoregulation, NKA subunit isoforms and NHE3 which are pump-mediated proteins, NKCC subunit isoforms which is a carrier-mediated protein and AQP isoforms which is a channel-mediated protein were selected to be investigated in terms of gene expression. Also, gene expression of other proteins was explored which are in the interaction network to which the aforementioned proteins belong.

As the salinity increased, the expression of NKA alpha and beta subunit isoforms slightly increased in the chum salmon fry gill. However, that of ATP1a1a decreased among the subunit isoforms. As mentioned above, the current study observed differences of the gene expression pattern within NKA subunit isoforms with increasing salinity. The same tendency was reported in previous studies of such euryhaline fishes as *Dicentrarchus labrax*, *O. mykiss*, *Oreochromis mossambicus*, *Salvelinus alpinus* and *S. salar*^34–37^. This indicates the possibility that either the ion transport activity or the absorption and secretion of ions varies within NKA subunit isoforms^36^. In this regard, two hypotheses have been reported: one, in the course of sodium and potassium transport, kinetics could vary due to the affinity difference between NKA subunit isoforms and ions, and the other that the concentration of lipid rafts rich in cholesterol and sphingolipids could affect the NKA subunit isoforms activity^38,39^. Specifically, in the case of the chum salmon fry gill, ATP1a1a was a predominant form in freshwater like other Salmonidae. As the salinity increased, however, ATP1a1b became a predominant form. The other NKA subunits (ATP1a, ATP1b isoforms) were found to have a somewhat lower effect on salinity change than ATP1a1a and ATP1a1b. Like ATP1a1a, gene expression of NHE3, an ion antiport protein, decreased with the increasing salinity. In other words, the sodium ion uptake activity was higher in freshwater than in seawater. This agrees with the research findings of studies of salinity and NHE3 expression in *D. labrax*, *O. mossambicus* and *Gasterosteus aculeatus*^40–43^. Another assumption is related to the sodium ion uptake and secretion. NHE3 and NKA present in gill mitochondria-rich cells (MRC) either uptake or secrete sodium ions. Hence, if the amount of sodium ion uptake decreases as the result of salinity increase, the amount of secretion will decrease as well. As a result, it is assumed that gene expression of ATP1a1a, a predominant form in freshwater fish, decreased with salinity increase.

In addition, it has been reported that rather than proton-pumping ATPase, a pump-mediated protein, NHE3 was activated in a yolk-sac membrane to balance hydrogen in the freshwater environment^42^. When they were transferred from freshwater to brackish water, the gill of chum salmon fry expressed more CFTR than when transferred from brackish water to seawater. That is, CFTR was greatly expressed when chum salmon fry were first exposed to salt stress. A similar pattern was observed in the gill of *F. heteroclitus*. It is known that Cl-secretion of CFTR in epithelial cells is controlled by adjusting the number of CFTR channels. This is known to be the result of the activation of the protein kinase A (PKA) caused by cAMP. Aquaporin, a channel-mediated protein, which is characterized by passive diffusion, was involved in the central nervous system (CNS) in cases of AQP4, AQP8 and AQP9 used in this study^44^. On the basis of functional features, AQP4 and AQP8 are classified as water-permeable aquaporin and AQP9 as aquaglyceroporin or permeable to water, glycerol and urea and AQP8 as permeable to water and urea^45^. Additionally, AQP8 and AQP9 are known to have ammonia transport capabilities^46^.

In this study, in the gill of chum salmon fry, AQP8 and AQP9 were expressed to a great extent when the fish were transferred from freshwater to brackish water. According to the findings of the studies of AQPs in *F. heteroclitus*, *Lateolabrax maculatus*, *O. nerka* and *S. salar*, AQP8 and AQP9 are rarely expressed in gills under the normal condition. The performance of qRT-PCR confirmed that the same was true for chum salmon fry in which AQP8 and AQP9 were hardly expressed in the gill of chum salmon fry in freshwater^47–50^. However, gene expression of AQP8 and AQP9 in the gill of chum salmon fry sharply increased with salinity increase. Likewise, gene expression of the AQP8 and AQP9 increased in the intestine of *A. japonica* and *O. nerka* in seawater in previous studies^47,51^. However, what underlies the expression pattern has not been clearly found so far. There are two possibilities: one that AQP8 and AQP9 would be expressed to secret ammonium and the other that the sudden movement of water molecules would cause the expression increase for the ion balance in and out of the body. As the salinity increases, the concentration of ammonium increases simultaneously, causing the secretion of ammonium in the body of the fish. In the process, the gill is reported to be involved in the secretion^52,53^.

Gene expression of AQP4 decreased with salinity increase and then increased again to control the cell-volume. This was observed in the case of transient receptor potential cation channel subfamily V member (TRPV4) which was present in the same interaction network as AQP4. However, the fold change value of TRPV4 was much lower than that of AQP4^54^. In previous research, AQP4 was expressed the most in the gill tissue of *Eptatretus burgeri* in the process of seawater adaptation, confirming the understanding that water transport is facilitated by an osmotic gradient in the gill^55^. That was in line with the findings of the current study. Referring to the PPI network analysis, transmembrane proteins were divided into five groups: NKA subunit isoforms group, AQP group, NKCC, CFTR and NHE3 group. The interaction between the groups can be seen on the map. NKCC1 and NKCC2 expressed in the gill of chum salmon fry were in the interaction network in which lysine deficient protein kinase (WNK), a chloride ion protein, was present. Also, both WNK3 which was ‘with no lysine’ family of serine-threonine protein kinase and NKCC1 which was in the same interaction network as STK39 (=SPAK) had the same expression pattern in chum salmon fry in the case of salinity increase. This was a similar tendency as seen in the WNK signaling pathway. Among NKA subunit isoforms, ATP1a1a had a similar expression pattern with FXYD6 which was a small membrane protein affecting gene expression of NKA alpha and beta complex. This agrees with the findings of a study on rats that discovered that FXYD6 was co-localized with NKA and FXYD6 bordered epithelial cells^56^. In addition to this, further research on the interaction network of various membrane proteins and analyses of gene expression patterns are expected to provide valuable information to research on functions of osmoregulatory proteins of fish.

Of special note is that while most of the fishes studied in other relevant research were in the smolt or the adult stage, the chum salmon used in this study were in the fry stage. Interestingly, the chum salmon fry showed a similar expression tendency to other fishes in the smolt or the adult stage although the expression of some essential membrane protein genes NKA, NKCC, NHE3, CFTR, and AQP which are involved in osmotic pressure control showed different patterns. The findings of this study confirm that chum salmon have excellent seawater adaptability early on, even in their fry stage. In addition, the comparative analysis of the gene expression patterns of the freshwater to brackish water group and the brackish water to seawater group indicates that 66% of the genes analyzed showed different expression patterns in both groups. In this respect, the types and the expression trends of various genes involved in balancing the body with a rapid increase in various ions were revealed.

This study was conducted on chum salmon, the species with the best osmotic control among salmonid fishes. It is expected that investigating the membrane proteins expressed in the gill of *O. keta*, which has a seawater acclimation ability even in the fry stage and studying the interaction network of membrane proteins and other various genes will contribute to future research on the osmoregulation pathway.

## Materials and Methods

### Ethics statement

All the experimental procedures with all the fish were performed and approved according to the guidelines of the Institutional Animal Care and Use Committee (IACUC) of Gangneung-Wonju National University (GWNU-2019-21). Furthermore, all the authors of this study have completed Animal Welfare & Ethics Course certification under the CITI program, research ethics and compliance training program.

### Salinity challenge and sample collection

The chum salmon at the alevin stage, one month after hatching, were transferred from the Korea Fisheries Resources Agency (FIRA) to the laboratory and reared in tanks with re -circulating freshwater for two months. Water temperature was maintained at 12 ± 1 °C. Aeration was provided continuously to maintain dissolved oxygen levels at 9.0 ± 0.5 mg/L. Fish were fed daily with commercial pellets and blood worms and fasted for 24 hours prior to the experiment.

In order to establish a stable seawater acclimation method, preliminary experiments were conducted by varying the salt concentration and the acclimation period. When the chum salmon fry were transferred from freshwater to seawater, most of the fry died within 20 days after the transfer. However, most of the chum salmon fry adapting to brackish water prior to moving to seawater survived approximately 20 days after the transfer. Based on these experiments, a methodology was established in which the individuals showed high survival rates within a short seawater acclimation period. No mortality was observed in the experimental group during salinity exposure. For the hyperosmotic challenge, fish (fry, average body weight and length = 0.6 ± 0.12 g and 4.87 ± 0.23 cm) were directly transferred to brackish water (50% seawater;17.5 ppt) for one day and acclimated in 100% seawater (35 ppt) for one day. Sampling (N = 5 in each group) was conducted at the same time points for the challenge. The 300 ppm of 2-phenoxyethanol (Sigma Aldrich Co, St. Louis, USA) was used as an anesthetic for sampling and gill tissues were extracted and stored at −80 °C.

### Library construction and illumina sequencing

Total RNA was extracted from the gill using RNAiso Reagent (Takara Bio, Shiga, Japan) according to the manufacturer’s instructions. Further purification was preceded by RNeasy Plus Mini Kit (Qiagen, Hilden, Germany) and RNase-free DNase set (Qiagen). The RNA-seq libraries were constructed using the Truseq stranded mRNA prep kit (Illumina, San Diego, Calif., USA). They were sequenced with a 2 × 101 bp (paired-end) read module using the Illumina Hiseq. 2500 platform. The raw sequencing files were generated using the Illumina base-calling software (CASAVA v1.8.2 with ASCII Q-score offset 33). Read-through adapter sequences, low-quality sequences (limit = 0.05), ambiguous nucleotides (maximal 2 nucleotides) were removed using CLC Genomics Workbench 11.0 (CLC Bio, QIAGEN) (Table S2).

### Genome mapping and *de-novo* assembly of unmapped reads

**Figure 6:**
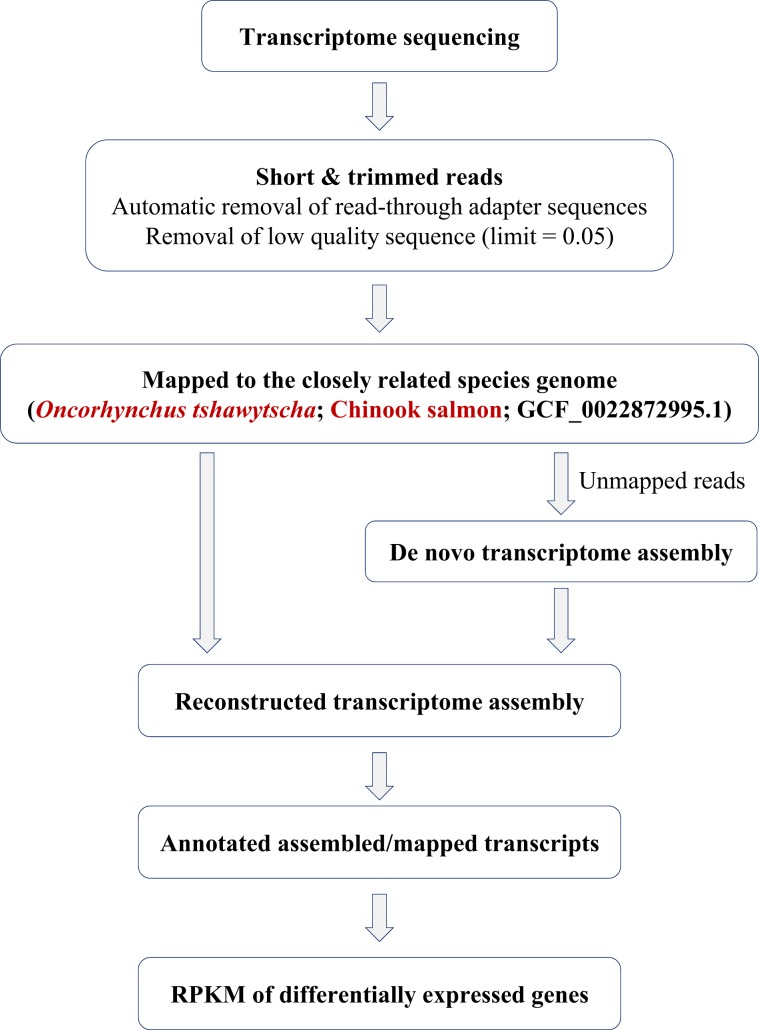
Schematic diagram of the transcriptome construction and the pipeline for annotation.

A schematic representation of the RNA-seq reference reconstitution and analysis pipeline is shown in Fig. 6. The chum salmon used in this study had no genome assembly information available as a reference. However, there was genome assembly information for chinook salmon (*O. tshawytscha*, GenBank assembly accession: GCF_002872995.1), which is taxonomically close to chum salmon among *Oncorhynchus* spp^57^. Trimmed sequences of each group were mapped to the chinook salmon reference. In addition, unmapped reads in the genome sequence of chinook salmon were *de-novo* assembled using CLC Genomics Workbench 11.0 under conditions of Kmer size 45, bubble size 50 and minimum contig length of 150 bp. Therefore, a new reference was constructed by combining both *de-novo* assembly data and the reads mapped to the reference genome. Finally, the annotation process was performed by mapping the trimmed readings to the newly constructed reference. The mapping was set under the following conditions: mismatch cost = 2, insertion cost = 3, deletion cost = 3, length fraction = 0.5, similarity fraction = 0.8, Auto-detect paired distances.

### Annotation of reconstructed reference sequences and differential expressed genes analysis

The mapping of the reads was performed with BLASTx-based annotation using BLAST2GO PRO v 5.2.5. BLASTx homology searches were carried after dynamic translation against NCBI non-redundant protein sequences (NR) database using the default cut-off parameters of E-value, 1.0e-3 and the word size of BLAST parameters of 3. In addition to the functional annotation, InterProScan v 5.34–73.0^58^, Gene ontology (GO; http://geneontology.org), GO-Slim, EggNOG v 4.5.1^59^ were used and the results were merged with the BLASTx annotation. The read counts that were mapped to the reconstructed reference were normalized to reads per kilobase of transcript per million mapped reads (RPKM) as the expression values. The false discovery rate (FDR) p-value less than 0.05 was used as a statistical value for differential expressed genes (DEG) screening and classified it into four groups of up-regulation (FC ≥ 1.5), moderately up-regulation (1 < FC < 1.5), down-regulation (FC ≤ −1.5) and moderately down-regulation (−1.5 < FC < −1) based on fold change (FC), respectively.

### Investigation and analysis of protein-protein interaction (PPI) networks

To investigate the expression of the membrane protein genes, the types of the proteins related to the network of the interaction among the membrane protein genes, analyses were conducted based on zebra fish (*Danio rerio*) database in STRING v11.0 (https://string-db.org/) on the condition of minimum required interaction score >0.5 and active interaction sources were as follows: Textmining, Experiments, Databases, Co-expression, Neighborhood, Gene Fusion, and Co-occurrence. Of the representative osmoregulatory membrane proteins, ATPase transporters (ATP1a1a, ATP1a1b, ATP1a3, ATP1b1, CFTR), symporters (SLC12a1, SLC12a2), antiporter (SLC9a3) and passive transporters (AQP4, AQP8, AQP9) were selected to be analyzed, and the expression of the various protein genes known to be related to the interaction network was investigated. Finally, an interaction networks map was completed on the multiple proteins used in the analyses and visualized with Cytoscape v3.7.1 software^60^.

### RNA-seq data validation by quantitative real-time RT-PCR (qRT-PCR)

The qRT-PCR was performed to verify expression patterns of differential expression genes in representative membrane protein genes of ionocytes as a result of RNA-Seq data. For qRT-PCR validation, normalization of the total RNA concentration between groups was performed, and cDNA was synthesized using PrimeScript RT reagent kit (Takara) with random primer and oligo-dTs. Primer design for qRT-PCR validation was based on the trimmed reads of chum salmon transcriptome sequencing. Specific primer pairs for membrane protein genes, including elongation factor 1 alpha as a qRT-PCR reference gene^61^ were constructed based on RNA-seq results (Table S3). qRT-PCR was conducted using a Thermal Cycler Dice ™ real-time PCR system (Takara) and SYBR premix Ex TaqII Kit (Takara). The qRT-PCR was carried out in triplicate on each sample. The thermal cycling was performed as follows: denaturation at 95 °C for 30 s, followed by 45 cycles of 95 °C for 5 s, and annealing at 60 °C for 30 s. qRT-PCR results were expressed as mean ± standard error (SEM) and performed with one-way ANOVA with significant level p < 0.05 using SPSS 25.0 software.

## Supplementary information

Dataset.

## References

[1] Kolar CS, Lodge DM (2002). Ecological predictions and risk assessment for alien fishes in North America. Science.

[2] Lee CE, Bell MA (1999). Causes and consequences of recent freshwater invasions by saltwater animals. Trends Ecol. Evol..

[3] Whitehead A (1999). The evolutionary radiation of diverse osmotolerant physiologies in killifish (*Fundulus* sp.). Evolution.

[4] Brennan RS, Galvez F, Whitehead A (2015). Reciprocal osmotic challenges reveal mechanisms of divergence in phenotypic plasticity in the killifish *Fundulus heteroclitus*. J. Exp. Biol..

[5] Folmar LC, Dickhoff WW (1980). The parr—smolt transformation (smoltification) and seawater adaptation in salmonids: A review of selected literature. Aquaculture.

[6] Esteve M, McLennan DA (2007). The phylogeny of *Oncorhynchus* (Euteleostei: Salmonidae) based on behavioral and life history characters. Copeia.

[7] Hoar WS (1988). 4 The physiology of smolting Salmonids. Fish Physiol. Biochem..

[8] Sundell K, Dellefors C, Björnsson BT (1998). Wild and hatchery-reared brown trout, *Salmo trutta*, differ in smolt related characteristics during parr–smolt transformation. Aquaculture.

[9] Black VS (1951). Changes in body chloride, density, and water content of chum (*Oncorhynchus keta*) and coho (*O. kisutch*) salmon fry when transferred from fresh water to sea water. Can. J. Fish. Aquat. Sci..

[10] Hasegawa S (1987). Osmoregulatory ability of chum salmon, *Oncorhynchus keta*, reared in fresh water for prolonged periods. Fish Physiol. Biochem..

[11] Kaneko T, Watanabe S, Lee KM (2008). Functional morphology of mitochondrion-rich cells in euryhaline and stenohaline teleosts. Aqua-BioSci. Monogr..

[12] Kaneko T, Hasegawa S, Takagi Y, Tagawa M, Hirano T (1995). Hypo-osmoregulatory ability of eyed-stage embryos of chum salmon. Mar. Biol..

[13] Iwata M, Komatsu S (1984). Importance of estuarine residence for adaptation of chum salmon (*Oncorhynchus keta*) fry to seawater. Can. J. Fish Aquat. Sci..

[14] Iwata M, Hirano T, Hasegawa S (1982). Behavior and plasma sodium regulation of chum salmon fry during transition into seawater. Aquaculture.

[15] Uchida K, Kaneko T (1996). Enhanced chloride cell turnover in the gills of chum salmon fry in seawater. Zool. Sci..

[16] Evans DH (2008). Teleost fish osmoregulation: what have we learned since August Krogh, Homer Smith, and Ancel Keys. Am. J. Physio.l Regul. Integra1. Comp. Physiol..

[17] Hwang P-P, Lee T-H, Lin L-Y (2011). Ion regulation in fish gills: recent progress in the cellular and molecular mechanisms. Am. J. Physiol. Regul. Integr. Comp. Physiol..

[18] Takei, Y. & Hwang, P-P. *Homeostatic responses to osmotic stress* 207–249 (Fish physiology, 2016).

[19] Marshall, W. S. & Grosell, M.* Ion transport, osmoregulation, and acid-base balance* 177–230 (Fish physiology, 2005).

[20] Evans DH, Piermarini PM, Choe KP (2005). The multifunctional fish gill: dominant site of gas exchange, osmoregulation, acid-base regulation, and excretion of nitrogenous waste. Physiol. Rev..

[21] Dymowska AK, Hwang P-P, Goss GG (2012). Structure and function of ionocytes in the freshwater fish gill. Respir. Physio.l Neurobiol..

[22] Kwong RWM, Kumai Y, Perry SF (2014). The physiology of fish at low pH: the zebrafish as a model system. J. Exp. Biol..

[23] Tomy S (2009). Salinity effects on the expression of osmoregulatory genes in the euryhaline black porgy *Acanthopagrus schlegeli*. Gen. Comp. Endocrinol..

[24] Sampaio LA, Bianchini A (2002). Salinity effects on osmoregulation and growth of the euryhaline flounder *Paralichthys orbignyanus*. J. Exp. Mar. Bio. Ecol..

[25] Li J, Wang J, Yang L, Chen Y, Yang Z (2014). Changes in plasma osmolality and Na+/K+ ATPase activity of juvenile obscure puffer *Takifugu obscurus* following salinity challenge. Biochem. Syst. Ecol..

[26] Shui C (2018). Serum osmolality and ions, and gill Na^+^/K^+^-ATPase of spottedtail goby *Synechogobius ommaturus* (R.) in response to acute salinity changes. Aquac. Fish.

[27] Lin YM, Chen CN, Lee TH (2003). The expression of gill Na, K-ATPase in milkfish, *Chanos chanos*, acclimated to seawater, brackish water and fresh water. Comp. Biochem. Physiol. A. Mol. Integr. Physiol..

[28] Pedersen PBM, Hansen K, Houng DTT, Bayley M, Wang T (2014). Effects of salinity on osmoregulation, growth and survival in Asian swamp eel (*Monopterus albus*) (Zuiew 1793). Aquac. Res..

[29] Zydlewski GB, Zydlewski J (2012). Gill Na^+^, K^+^-ATPase of Atlantic salmon smolts in freshwater is not a predictor of long-term growth in seawater. Aquaculture.

[30] Hiroi J, McCormick SD (2012). New insights into gill ionocyte and ion transporter function in euryhaline and diadromous fish. Respir. Physiol. Neurobiol..

[31] Ishibashi, K., Morishita, Y. & Tanaka, Y. *The evolutionary aspects of aquaporin family* 35–50 (Aquaporins Springer, 2017). 10.1007/978-94-024-1057-0_2 28258564

[32] Marshall WS (2011). Mechanosensitive signaling in fish gill and other ion transporting epithelia. Acta. Physiol..

[33] Crespi BJ, Fulton MJ (2004). Molecular systematics of Salmonidae: combined nuclear data yields a robust phylogeny. Mol. Phylogenet. Evol..

[34] Bystriansky JS, Richards JG, Schulte PM, Ballantyne JS (2006). Reciprocal expression of gill Na^+^/K^+^-ATPase alpha-subunit isoforms alpha1a and alpha1b during seawater acclimation of three salmonid fishes that vary in their salinity tolerance. J. Exp. Biol..

[35] Nilsen TO (2007). Differential expression of gill Na^+^, K^+^-ATPase alpha- and beta-subunits, Na^+^, K^+^, 2Cl^−^ cotransporter and CFTR anion channel in juvenile anadromous and landlocked Atlantic salmon Salmo salar. J. Exp. Biol..

[36] McCormick SD, Regish AM, Christensen AK (2009). Distinct freshwater and seawater isoforms of Na^+^/K^+^-ATPase in gill chloride cells of Atlantic salmon. J. Exp. Biol..

[37] Tipsmark C (2011). Switching of Na^+^, K^+^-ATPase isoforms by salinity and prolactin in the gill of a cichlid fish. J. Endocrinol..

[38] Lin C-H, Lee T-H (2005). Sodium or potassium ions activate different kinetics of gill Na, K‐ATPase in three seawater‐and freshwater‐acclimated euryhaline teleosts. J. Exp. Zool. A. Comp. Exp. Biol..

[39] Lingwood D, Harauz G, Ballantyne JS (2005). Regulation of fish gill Na^+^-K^+^-ATPase by selective sulfatide-enriched raft partitioning during seawater adaptation. J. Biol. Chem..

[40] Blondeau-Bidet E, Hiroi J, Lorin-Nebel C (2019). Ion uptake pathways in European sea bass *Dicentrarchus labrax*. Gene.

[41] Gibbons TC, Metzger DCH, Healy TM, Schulte PM (2017). Gene expression plasticity in response to salinity acclimation in three-spine stickleback ecotypes from different salinity habitats. Mol. Ecol..

[42] Inokuchi M, Hiroi J, Watanabe S, Lee KM, Kaneko T (2008). Gene expression and morphological localization of NHE3, NCC and NKCC1a in branchial mitochondria-rich cells of Mozambique tilapia (*Oreochromis mossambicus*) acclimated to a wide range of salinities. Comp. Biochem. Physiol. A. Mol. Integr. Physiol..

[43] Watanabe S, Niida M, Maruyama T, Kaneko T (2008). Na^+^/H^+^ exchanger isoform 3 expressed in apical membrane of gill mitochondrion-rich cells in Mozambique tilapia *Oreochromis mossambicus*. Fish Sci..

[44] Barbara B (2010). Aquaporin biology and nervous system. Curr. Neuropharmacol..

[45] King LS, Kozono D, Agre P (2004). From structure to disease: the evolving tale of aquaporin biology. Nat. Rev. Mol. Cell Biol..

[46] Litman T, Søgaard R, Zeuthen T (2009). Ammonia and urea permeability of mammalian aquaporins. Handb. Exp. Pharmacol..

[47] Choi YJ (2013). Expression of aquaporin-3 and −8 mRNAs in the parr and smolt stages of sockeye salmon, *Oncorhynchus nerka*: Effects of cortisol treatment and seawater acclimation. Comp. Biochem. Physiol. A. Mol. Integr. Physiol..

[48] Jung D (2015). A novel variant of aquaporin 3 is expressed in killifish (*Fundulus heteroclitus*) intestine. Comp Biochem Physiol C: Toxicol. Pharmacol..

[49] Shen Y (2019). Transcriptome analysis of gill from *Lateolabrax maculatus* and aqp3 gene expression. Aquac. Fish.

[50] Tipsmark CK, Sørensen KJ, Madsen SS (2010). Aquaporin expression dynamics in osmoregulatory tissues of Atlantic salmon during smoltification and seawater acclimation. J. Exp. Biol..

[51] Kim YK, Watanabe S, Kaneko T, Huh MD, Park SI (2010). Expression of aquaporins 3, 8 and 10 in the intestines of freshwater-and seawater-acclimated Japanese eels *Anguilla japonica*. Fish Sci..

[52] Pradeep PJ (2011). Trend in ammonia excretion during acclimatization of adult freshwater red hybrid tilapia *Oreochromis mossambicus* (Peters, 1852) X *Oreochromis niloticus* (Linnaeus, 1758) in different salinities. Our Nature.

[53] Wood, C. & Evans, D. Ammonia and urea metabolism and excretion [in fish]. *CRC. Mar. Sci. Ser*. 379–426 (1993).

[54] Benfenati V (2011). An aquaporin-4/transient receptor potential vanilloid 4 (AQP4/TRPV4) complex is essential for cell-volume control in astrocytes. Proc. Natl. Acad. Sci. USA.

[55] Nishimoto G (2007). Molecular characterization of water-selective AQP (EbAQP4) in hagfish: insight into ancestral origin of AQP4. Am. J. Physiol. Regul. Integr. Comp. Physiol..

[56] Delprat B (2007). FXYD6 is a novel regulator of Na, K-ATPase expressed in the inner ear. Biol. Chem..

[57] Christensen KA (2018). Chinook salmon (*Oncorhynchus tshawytscha*) genome and transcriptome. PloS one.

[58] Finn RD (2017). InterPro in 2017-beyond protein family and domain annotations. Nucleic. Acids. Res..

[59] Huerta-Cepas J (2017). Fast genome-wide functional annotation through orthology assignment by eggNOG-mapper. Mol. Biol. Evol..

[60] Shannon P (2003). Cytoscape: a software environment for integrated models of biomolecular interaction networks. Genome Res..

[61] Olsvik PA, Lie KK, Jordal AO, Nilsen TO, Hordvik I (2005). Evaluation of potential reference genes in real-time RT-PCR studies of Atlantic salmon. BMC. Mol. Biol..

