# Supplementary material for: Comparative transcriptome profiling of selected osmotic regulatory proteins in the gill during seawater acclimation of chum salmon (Oncorhynchus keta) fry
Source: Sci Rep. 2020 Feb 6;10:1987. doi: 10.1038/s41598-020-58915-6 (PMC7005315; doi:10.1038/s41598-020-58915-6)
Supplement: Supplementary file 1 — Dataset. [file 41598_2020_58915_MOESM1_ESM.pdf]

Supporting information for

Comparative transcriptome profiling of selected osmotic regulatory proteins in the gill during seawater acclimation of chum salmon (*Oncorhynchus keta*) fry

Sang Yoon Lee[a], Hwa Jin Lee[b] and Yi Kyung Kim[a,b]

[a]The East Coast Research Institute of Life Science, Gangneung-Wonju National University, Gangneung 25457, South Korea

[b]Department of Marine Biotechnology, Gangneung-Wonju National University, Gangneung 25457, South Korea

*Corresponding author: Yi Kyung Kim (yikyung1118@gwnu.ac.kr)

Figure S1. Species distribution of the BLASTx top hit annotation.
Figure S2. Gene ontology (by level 7) classification of *O. keta* fry gill transcriptome.
Table S1. Mapping statistics of transcriptome reads to the *O. tshawytscha* genome assembly.
Table S2. Statistics of transcriptome sequencing data used in this study.
Table S3. Primer information of qRT-PCR validation in this study.

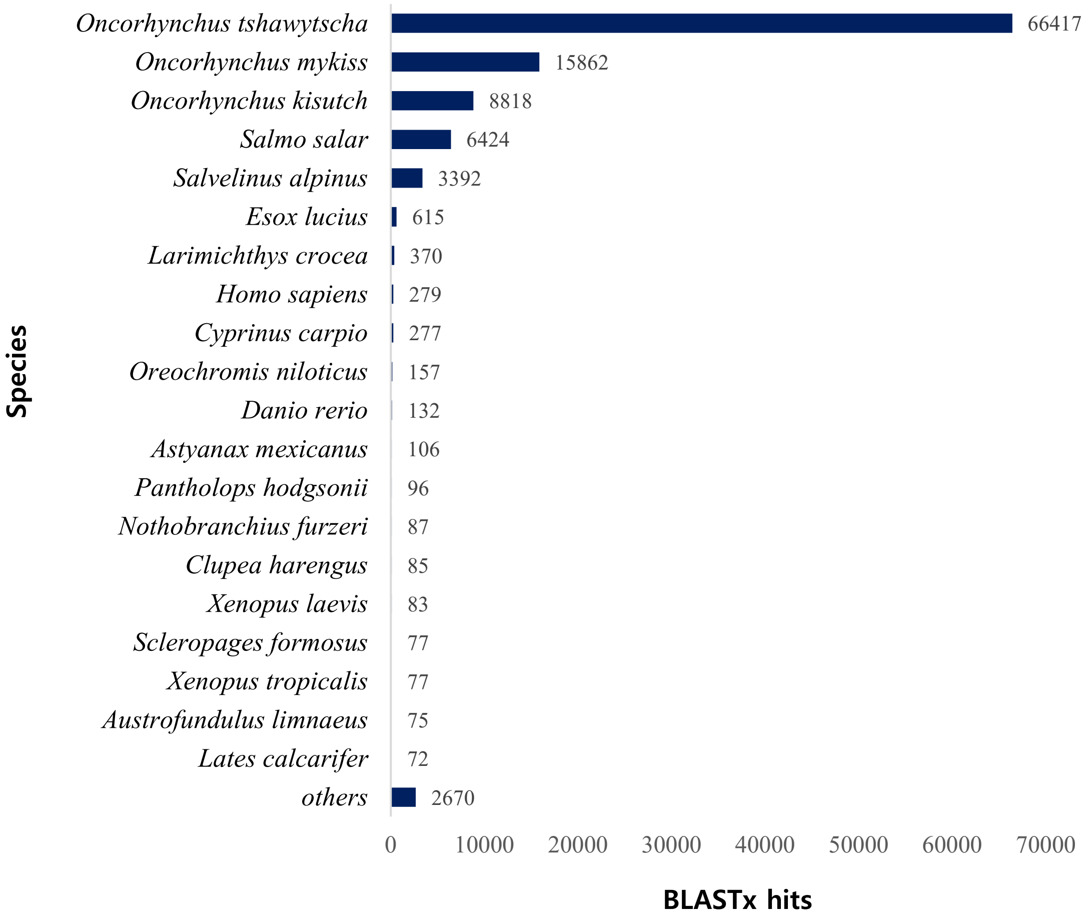

Figure S1. Species distribution of the BLASTx top hit annotation.

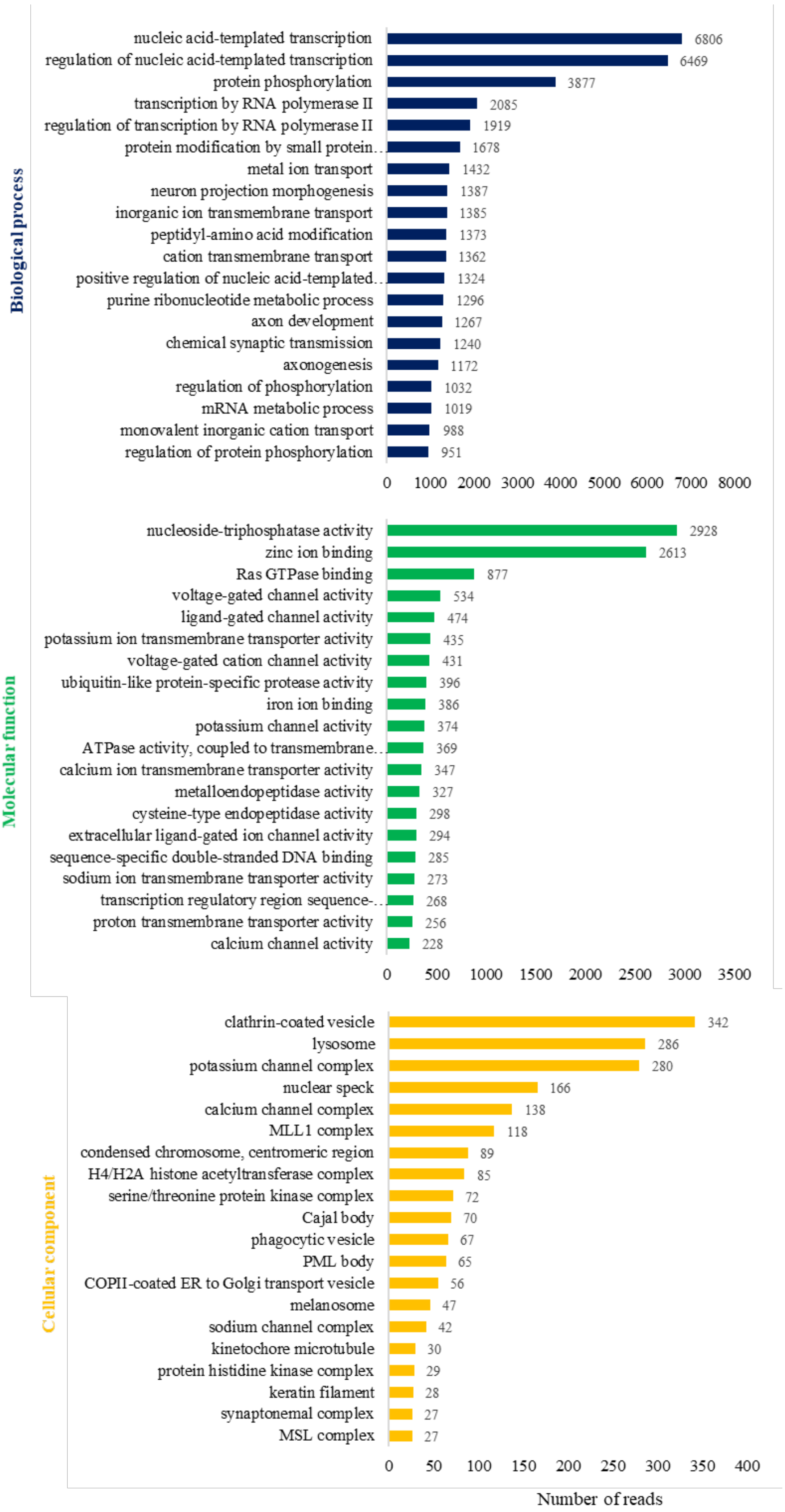

Figure S2. Gene ontology (by level 7) classification of *O. keta* fry gill transcriptome.

Table S1. Mapping statistics of transcriptome reads to the *O. tshawytscha* genome assembly.

	Fresh water (0%)		Brackish water (50%)		Sea water (100%)	
	No. reads	Rate (%)	No. reads	Rate (%)	No. reads	Rate (%)
Mapped reads	80,882,718	82.73	79,861,424	82.18	91,925,280	82.59
Not mapped reads	16,880,700	17.27	17,315,810	17.82	19,375,610	17.41
Reads in pairs	65,491,816	66.99	64,289,650	66.16	73,879,228	66.38
Broken paired reads	15,390,902	15.74	15,571,774	16.02	18,046,052	16.21
Total reads	97,763,418	100.00	97,177,234	100.00	111,300,890	100.00

Table S2. Statistics of transcriptome sequencing data used in this study.

Sample name	Number of reads	Average length (bp)	Number of reads after trim	Percentage trimmed (%)
Freshwater (0% salinity)	100,704,826	101	97,177,234	96.50
Brackish water (50% salinity)	101,802,138	101	97,763,418	96.03
Seawater (100% salinity)	116,438,490	101	111,300,890	95.59

Table S3. Primer information of qRT-PCR validation in this study.

Genes	Primers	Sequences (5′ - 3′)
ATP1a1a	qOK_ATP1a1a_1F	TGGCCGAACAGAACAACGTA
	qOK_ ATP1a1a_1R	TGGAGTTGAAGGGGATCTCA
ATP1a1b	qOK_ ATP1a1b_1F	GCAGGGAATGAAGAACCGTA
	qOK_ ATP1a1b_1R	GGTTTCGTCGCAGGATGTAT
ATP1a1c	qOK_ ATP1a1c_1F	TCCTTGCTGAGAATGGGTTC
	qOK_ ATP1a1c_1R	TACTGGCGAAGAATGCTGTG
ATP1a3	qOK_ATP1a3_1F	ATCAGGCTAAACTGGGACGA
	qOK_ATP1a3_1R	CCTGGTCTTGCAGATGATGA
ATP1b1	qOK_ATP1b1_1F	CCAATGAAAGCATCCCTGAG
	qOK_ATP1b1_1R	TGAAGCAGCTTGCCGTAGTA
SLC12a1	qOK_SLC12a1_1F	CACCCAAAACCCCAACTCTA
	qOK_SLC12a1_1R	CTTTTCCTCGAAGGTGTCCA
SLC12a2a	qOK_SLC12a2a_1F	TCAACAGGATTGACCACGAC
	qOK_SLC12a2a_1R	TGCTGTATCCTGCTCCATGT
SLC12a2b	qOK_SLC12a2b_1F	GTGGAAGGACTGCAAAATCC
	qOK_SLC12a2b_1R	TACGGCTCGATCATTTCCTC
CFTR	qOK_CFTR_1F	TCCTGAGGAAGACCCTGAAA
	qOK_CFTR_1R	TGGCTGGTCTCATTCAACAG
SLC9a3	qOK_SLC9a3_1F	CCATGCTTATGGGGATGAAG
	qOK_SLC9a3_1R	AGCCATGAAGGAATCTGCTG
AQP4	qOK_AQP4_1F	AGTTCTTCTTTGCCCCCAGT
	qOK_AQP4_1R	TGCTCCTGTTTGTTGACCTG
AQP8	qOK_AQP8_1F	GGGAGCATTCAACGTGGTTA
	qOK_AQP8_1R	ACCCGCCAGCCAGTATATTA
AQP9	qOK_AQP9_1F	CCATCCATATCGGCTTCACT
	qOK_AQP9_1R	GACAGCACAAGATCCAGCAA
EF1α	qOK_EF1α_1F	AGCTCAAGGAGAAGATCGAC
	qOK_EF1α_1R	CTTGATGACACCAACAGCCA
